# The C-Terminal Domains of NF-H and NF-M Subunits Maintain Axonal Neurofilament Content by Blocking Turnover of the Stationary Neurofilament Network

**DOI:** 10.1371/journal.pone.0044320

**Published:** 2012-09-21

**Authors:** Mala V. Rao, Aidong Yuan, Jabbar Campbell, Asok Kumar, Ralph A. Nixon

**Affiliations:** 1 Center for Dementia Research, Nathan Kline Institute, Orangeburg, New York, United States of America; 2 Department of Psychiatry, New York University Langone Medical Center, New York, New York, United States of America; 3 Department of Cell Biology, New York University Langone Medical Center, New York, New York, United States of America; 4 Department of Pathology, New York University Langone Medical Center, New York, New York, United States of America; Stanford University School of Medicine, United States of America

## Abstract

Newly synthesized neurofilaments or protofilaments are incorporated into a highly stable stationary cytoskeleton network as they are transported along axons. Although the heavily phosphorylated carboxyl-terminal tail domains of the heavy and medium neurofilament (NF) subunits have been proposed to contribute to this process and particularly to stability of this structure, their function is still obscure. Here we show in NF-H/M tail deletion [NF-(H/M)^tailΔ^] mice that the deletion of both of these domains selectively lowers NF levels 3–6 fold along optic axons without altering either rates of subunit synthesis or the rate of slow axonal transport of NF. Pulse labeling studies carried out over 90 days revealed a significantly faster rate of disappearance of NF from the stationary NF network of optic axons in NF-(H/M)^tailΔ^ mice. Faster NF disappearance was accompanied by elevated levels of NF-L proteolytic fragments in NF-(H/M)^tailΔ^ axons. We conclude that NF-H and NF-M C-terminal domains do not normally regulate NF transport rates as previously proposed, but instead increase the proteolytic resistance of NF, thereby stabilizing the stationary neurofilament cytoskeleton along axons.

## Introduction

Neurofilaments (NFs) are neuron-specific 10-nm intermediate filaments essential for radial growth of axons [1], [2], [3], [4], and efficient propagation of electric impulses along axons [5]. Various properties of NF composition, structure and dynamic behavior have been proposed to influence the accumulation of NF along axons that underlies caliber expansion and may determine shapes of other regions of the neuron. To achieve this stable geometry, axonally transported NF contribute to a large stationary cytoskeletal network, which also serves as a scaffold for the reversible docking of organelles and proteins, thereby regulating their activity, abundance, and trafficking [6]. In serving these roles, different subunits of the NF bind to specific molecular motors, receptor proteins, and other cytoskeletal proteins [7], [8], [9].

NFs are obligate heteropolymers [10], [11] composed of neurofilament heavy (NF-H; 200 kDa), medium (NF-M; 160 kDa), low (NF-L; 68 kDa) and α-internexin in CNS axons [12]. The exceptionally long NF-H and NF-M carboxyl terminal tail domains contain 51 and 7 phosphorylation sites, respectively within repeated serine-lysine-proline (KSP) sequences [13], which are regulated by multiple protein kinases (ERK 1/2, JNK, and cdk5) and multiple phosphatases [14], [15], [16]. C-terminal domain phosphorylation straightens, aligns, and bundles NFs and extends C-terminal sidearms *in vitro* [17] promoting cross bridge formation among NF and other cytoskeletal elements [18], and an increase in inter-filament spacing [19], [20], [21], [22]. The acquisition of phosphates on the C-terminal domains occurs mainly after NFs enter axons and coincides with the integration of NF undergoing slow transport into a long-lived stationary cytoskeletal network containing interconnected NF, microtubules (MT), actin filaments [23], [24], and various cytoskeletal cross linker proteins [23], [25], [26], [27], [28]. These events are associated with local NF accumulation and axon caliber expansion [19], which are, in turn, triggered by signals from myelinating glia [29].

Observations that abnormal degrees of hyperphosphorylation of NF-H and NF-M C-terminal domains can slow NF transport [30], [31], [32] and that the NF proteins in their highest states of phosphorylation are present in the stationary NF network [26] has suggested that C-terminal phosphorylation might regulate the rate of NF transport, although this suggestion is at odds with recent studies that deletion of either the C-terminal domain of NF-H or NF-M does not change NF transport rates [21], [22], [33], [34]. It is possible, however, that effects on transport might arise only if both tail domains are missing given that NF-H and NF-M tail domains share structural similarities and could be functionally redundant [22], [33]. To test this hypothesis and further examine the roles of these C-terminal domains in regulating axon architecture, we generated mice that lack carboxyl terminal domains of NF-H and NF-M subunits. Our studies with these mice unequivocally demonstrate that C-terminal domain deletion does not alter NF transport rate but rather decreases the stability of NFs in the stationary cytoskeleton. The profound reduction of steady-state axonal NF content after tail domain deletion and the rise in proteolytic fragments of NF-L protein implicate the tail domains of NF-M and NF-H as major determinants of the high proteolytic resistance and exceptionally long half-lives of NF and, thus, as key regulators of NF number in axons.

## Methods

### Ethics Statement

All the animal protocols described in the paper were approved by the Nathan Kline Institute Animal Care and Use Committee Protocol AP2005-155.

### Production of NF-(H/M)^tailΔ^ mice

The male mice that are heterozygous for NF-M^tailΔ^ [22] and female mice for NF-H^tailΔ^ [21] were bred to produce NF-(M/H)^tailΔ^, and their littermate controls at a ratio of 1∶16 respectively, with NF-H^tailΔ^, and NF-M^tailΔ^ mice. The genomic tail DNAs isolated from the mice was separately screened for NF-M^tailΔ^ according to Rao et al., 2003 [22] and for NF-H^tailΔ^ according to Rao et al., 2002a [21].

### Immunoblotting of full length and tail deleted neurofilament subunits

Sciatic and optic nerve extracts were made from 6 month old animals as described previously [35]. Protein concentration was determined using bicinchoninic acid (BCA) assay kit (Pierce Chemical Co., Rockford, IL). Protein extracts, as well as known amounts of neurofilament standards, were separated on 7% polyacrylamide gels with SDS and transferred to nitrocellulose membranes. Full length and the NF-M^tailΔ^ proteins were detected with a monoclonal antibody raised against the rod domain of NF-M subunit (mAb, RMO44, Zymed, Alameda, CA). The NF-H (pAb-NF-H_COOH_, [35]), NF-L (mAb NR-4, Sigma, St. Louis, MO), the Myc tagged NF-H^tailΔ^ and NF-M^tailΔ^ subunits with a Myc antibody (mAb, 9E10, Santa Cruz Biotech, CA), R-39 (Dr. Doris Dahl), calnexin (StressGen Biotech, Canada), α-tubulin, β-tubulin, and neuron-specific β_III_-tubulin are from Sigma, St. Louis, MO. After blots were washed, incubated with appropriate secondary antibodies conjugated with HRP and developed with ECL reagent, immunoreactive bands were visualized by autoradiography and quantified by phosphorimaging (Molecular Dynamics) using known amounts of purified mouse spinal cord neurofilament standards.

For the analysis of NF subunits along optic nerves, optic pathways from NF-(H/M)^tailΔ^ and Wild Type mice at 6 months of age were cut into 1 mm segments, and protein extracts were made as described previously [35]. Equal volumes of extracts were resolved on 7% polyacrylamide gels with SDS, transferred to nitrocellulose membranes, some of the blots were Ponceau S stained and other blots were immunoblotted with antibodies as described above.

### Axonal transport studies

Retinal ganglion cells from 3–4 month old NF-(H/M)^tailΔ^ or their WT littermate mice were radiolabeled *in situ* with 100 µCi of [^35^S]-methionine by intravitreal injection with a calibrated micropipette apparatus into anesthetized mice [36]. After injection, mice were sacrificed by cervical dislocation, and optic pathways were dissected after 3 days, one, and two weeks. Three animals were analyzed for each genotype and time point. The optic pathways were frozen and cut into 8 consecutive segments of each 1 mm. Each segment was homogenized with a buffer containing 1% Triton X-100, 50 mM Tris, pH 6.8, 2 mM EDTA, 1 mM PMSF, and 50 µg/ml of protease inhibitor cocktail (Boehringer Mannheim). After centrifugation, the Triton insoluble cytoskeleton and soluble protein fractions were analyzed on 5–15% polyacrylamide gradient gels, transferred to nitrocellulose membranes and quantified by phosphorimaging.

### Synthesis and Turnover of NF-L

To measure the synthesis of NF-L in retinal ganglion cells of WT and NF-(H/M)^tailΔ^, mice were injected intravitreously with [^3^H]-proline and sacrificed after 14 days. Optic pathways were collected, fractionated as indicated above, NF-L bands on Coomassie stained gels were cut out and radioactivity in the appropriate bands was quantified in scintillation fluid. To measure the turnover of NF-L, groups of 30 WT and NF-(H/M)^tailΔ^ mice were intravitreously injected with the same amount of [^3^H]-proline and quantified as described previously [37]. Briefly, after injection, 15 mice from each WT and NF-(H/M)^tailΔ^ group were sacrificed 14 days and at 90 days. Cytoskeletal preparations were made and fractionated as indicated above. Gels were stained with Coomassie Blue and the bands corresponding to NF-L were cut out and quantified. The counts obtained at 90 days over 14 days were calculated and expressed as the ratio of retained NF-L (turnover of NF-L) for both the genotypes. We routinely use [^3^H]-proline labeling for long term studies instead of [^35^S]-methionine labeling because [^35^S]-methionine has a much shorter half-life and is unsuited for long term labeling.

### Morphometric analysis

NF-(H/M)^tailΔ^ and their littermate WT mice of 6 month old were perfused transcardially with 4% paraformaldehyde, 2.5% glutaraldehyde in 0.1 M sodium cacodyalate buffer, pH 7.2, and post fixed overnight in the same buffer. Samples were treated with 2% osmium tetroxide, washed, dehydrated and embedded in Epon-Araldite resin. Thick sections at 50 µm and 2 mm region of optic nerve (0.75 µm) for light microscopy were stained with toluidine blue, and thin sections (70 nm) for electron microscopy were stained with uranyl acetate and lead acetate. Neurofilaments and microtubules were counted from 1000 axons for each genotype (n = 4), axon diameters were measured using the Bioquant Software (Nashville, TN). NF and microtubule densities were calculated as reported earlier [29], [38].

## Results

### Deletion of both NF-M and NF-H tail domains markedly lowers NF content of axons

**Figure 1:**
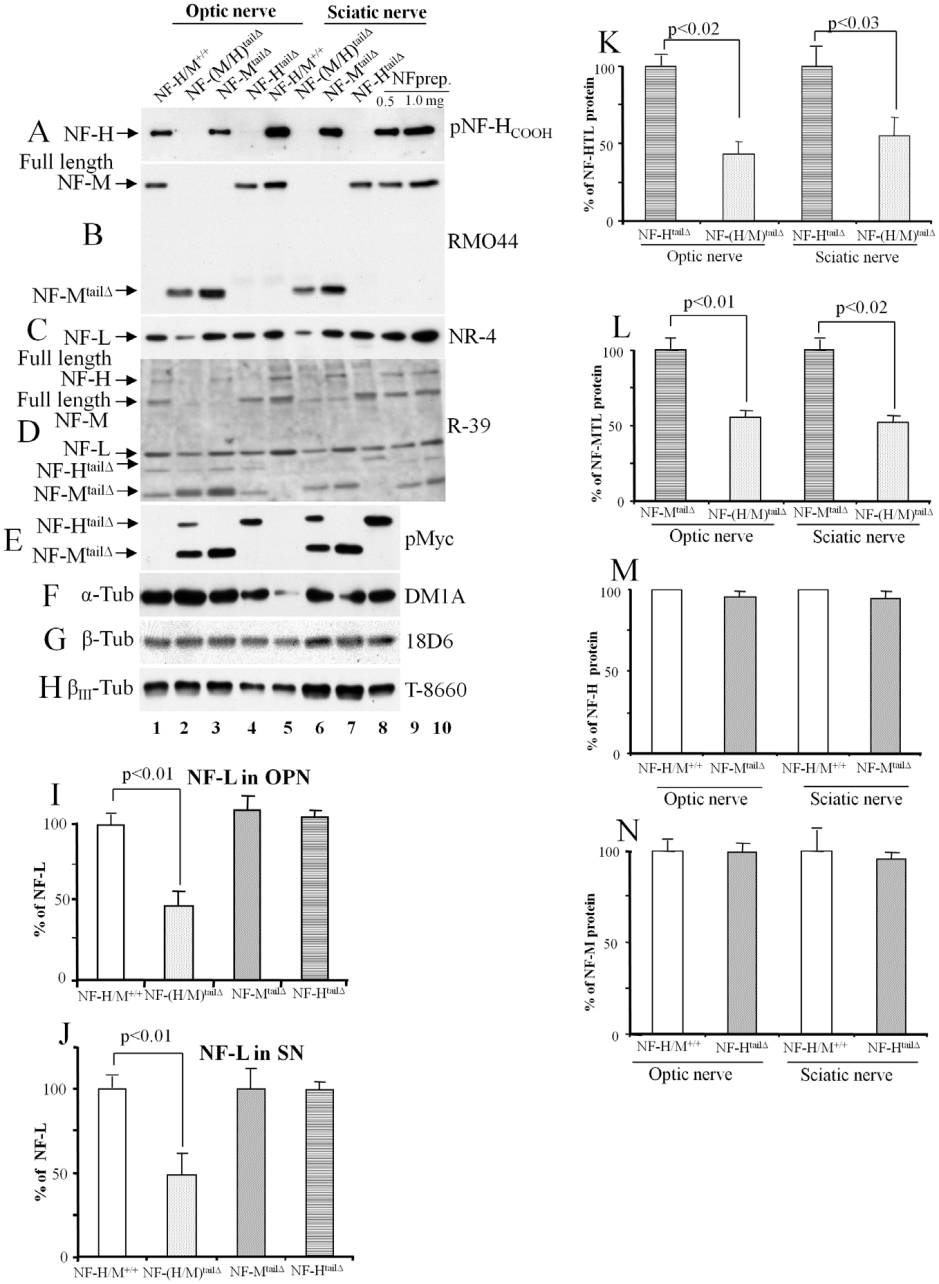
Loss of both the tails of NF-H/M subunits results in reduced neurofilament content in axons. Equal amounts of optic (lanes 1–4) and sciatic nerve (lanes 5–8) extracts were fractionated on 7% SDS-polyacrylamide gels, immunoblotted with NF-H (Fig. A), NF-M (Fig. B), NF-L (Fig. C), NF triplets (Fig. D), tail deleted NF-H and NF-M (Fig. E), α-tubulin (Fig. F), β-tubulin (Fig. G), and β_III_-tubulin (H) antibodies. Quantification of NF-L in optic (Fig. I), and sciatic nerves (Fig. J); NF-H^tailΔ^ (Fig. K); NF-M^tailΔ^ (Fig. L); full length NF-H (Fig. M), and full length NF-M (Fig. N). NF preparation from WT spinal cords indicates the position of full length NF subunits on immunoblots (lanes 9&10). Error bars represent SEM in all experiments.

Immunoblot analyses with a panel of NF antibodies [35] revealed that levels of each NF subunit were lowered >50% in optic nerves (lanes 1–4) and sciatic nerves (lanes 5–8) of NF-(H/M)^tailΔ^ mice (Fig. 1A–E). The markedly reduced NF-L levels in NF-(H/M)^tailΔ^ axons (Fig. 1I, J) were not seen in mice with single tail deletions (NF-H^tailΔ^ or NF-M^tailΔ^) (Fig. 1I–J). Similarly, the truncated NF-H and NF-M subunits were significantly reduced in NF-(H/M)^tailΔ^ mice (Fig. 1K, 1L), but these same tail-deleted subunits and the other full length subunits were unaltered in single tail-deleted mutants (Fig. 1I, J, M, N) as previously observed [21], [22]. By contrast, tubulin levels (α, β, and neuron specific β_III_ subunits) rose significantly in NF-(H/M)^tailΔ^ axons (Fig. 1F–H), as seen earlier in NF-M tail-less [22], but not in NF-H tail-less mice [21]. The findings were similar whether identical amounts of protein or nerve segments of equal length (data not shown) were analyzed on immunoblots.

**Figure 2:**
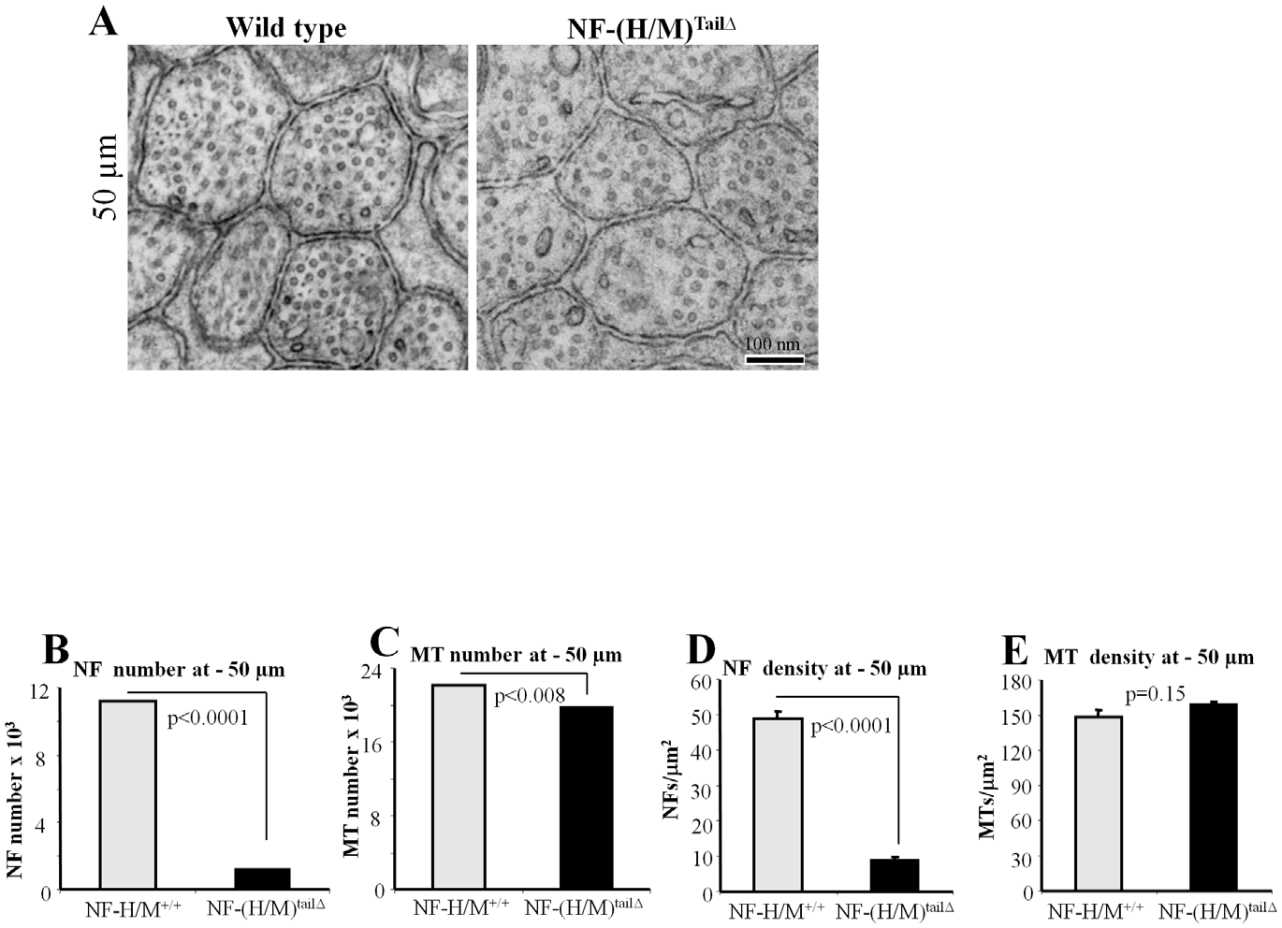
A marked reduction in NF number and density at 50 µm area of NF-(H/M)^tailΔ^ optic axons. Distribution of NFs and MTs in WT and NF-(H/M)^tailΔ^ optic axons at 50 µm (A). Bar: 100 nm. WT and NF-(H/M)^tailΔ^ optic nerves at 50 µm area analyzed for NF and MT number (B, C) and density (D, E). NF (B) and MT numbers (C) at 50 µm in WT and NF-(H/M)^tailΔ^ mice. NF (D) and MT density (E) at 50 µm area. Data is from WT-1000, and NF-(H/M)^tailΔ^ - 1000 axons. Error bars represent SEM in all experiments.

**Figure 3:**
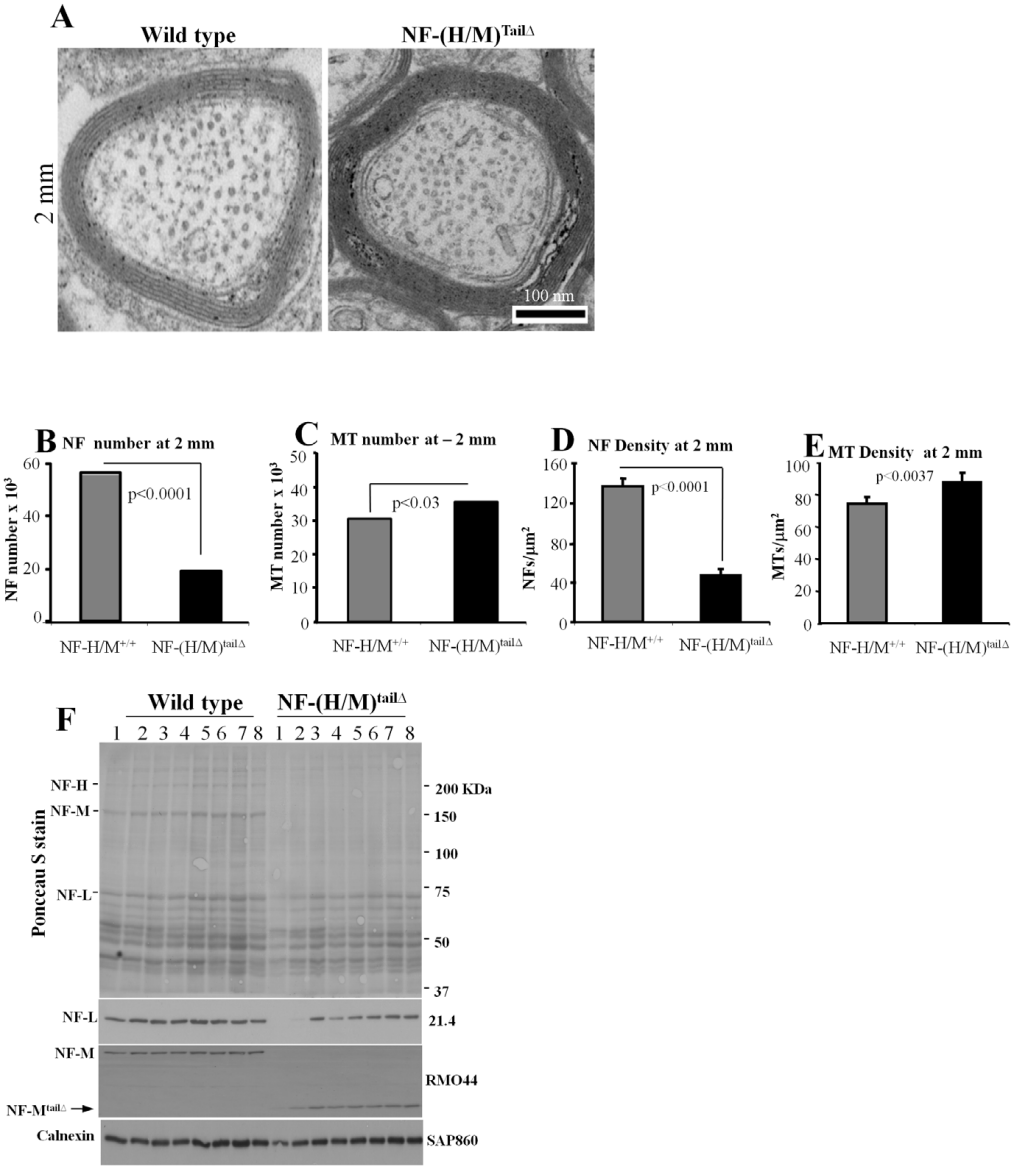
NF-M/H tails regulate NF integration, number, density and establishment of stationary NF networks in optic axons. Distribution of NFs and MTs in WT and NF-(H/M)^tailΔ^ optic axons at 2 mm region of optic nerve (A). Bar: 100 nm. WT and NF-(H/M)^tailΔ^ optic nerves at 2 mm area analyzed for NF and MT number (B, D) and density (C, E). NF (B) and MT numbers (C) at 2 mm in WT and NF-(H/M)^tailΔ^ mice. NF (D) and MT density (E) at 2 mm area of WT and NF-(H/M)^tailΔ^ optic axons. Data is from WT-1000, and NF-(H/M)^tailΔ^ - 1000 axons. (F). Integration of NFs along the optic axonal segments is reduced in NF-(H/M)^tailΔ^ optic axons. Calnexin staining is used as a loading control. Error bars represent SEM in all experiments.

To find out whether the reduction in NF subunit levels of NF-(H/M)^tailΔ^ axons by immunoblots is associated with reduction in NF number and density in axons, we examined the proximal region (50 µm area, Fig. 2) and the distal area (2 mm, Fig. 3) of optic nerve by electron microscopy. Our examination and quantification of NFs from electron micrographs indicate a marked drop in the numbers and density of NF at 50 µm from the retinal excavation (Fig. 2A–E, Table 1). We also measured the number and the density of NFs at 2 mm region of the NF-(H/M)^tailΔ^ optic nerves (Fig. 3A–E, Table 1) and find that NF number and density is reduced even at 2 mm region of the optic nerve (Fig. 3B, D). NF number and density in NF-(H/M)^tailΔ^ axons were 6-fold lower at the 50 µm level (Table 1) relative to values in WT mice and >3-fold lower at the 2-mm level (Table 1). By contrast, the number of microtubules, unlike total tubulin levels, was minimally altered (Fig. 2C, E & 3C, E). Analysis of consecutive 1 mm segments of optic nerve samples from NF-(H/M)^tailΔ^ mice demonstrates reduced NF-L staining. Protein bands above 75 kDa are also reduced on Ponceau S stained gels, due to loss of the most abundant full length NF-H and NF-M proteins on these gels (3F). Quantitative immunoblot analyses of consecutive 1 mm segments along the entire optic pathway revealed even greater NF subunit losses at proximal levels of the optic nerve than more distally in NF-(H/M)^tailΔ^ mice (e.g., NF-L, 70% proximally versus 50% distally) when expressed relative to levels of calnexin, which distributes uniformly along optic axons (Fig. 3F). These results demonstrate that NF-(H/M) tails are essential for establishment of stationary phase of NFs in the proximal area and normal integration of NFs all along the optic nerve.

**Table 1:** NF-H/M tails together regulate NF number and density in axons.

	Axons	Area µm	NFs	MTs	NFs-D	MTs-D
NF-H/M+/+ 50 µm	1000	212.6	**11200**	22055	52.7	103.7
NF-(H/M)^tailΔ^ 50 µm	1000	144.7	**1195**	19757	8.3	136.5
NF-H/M+/+ 2 mm	1000	414	**56563**	30547	136.6	73.8
NF-(H/M)^tailΔ^ 2 mm	1000	409	**19120**	35614	46.7	87.1

### Deletion of NF-M/H tail domains does not alter rates of entry or slow transport of newly synthesized NF in optic axons

**Figure 4:**
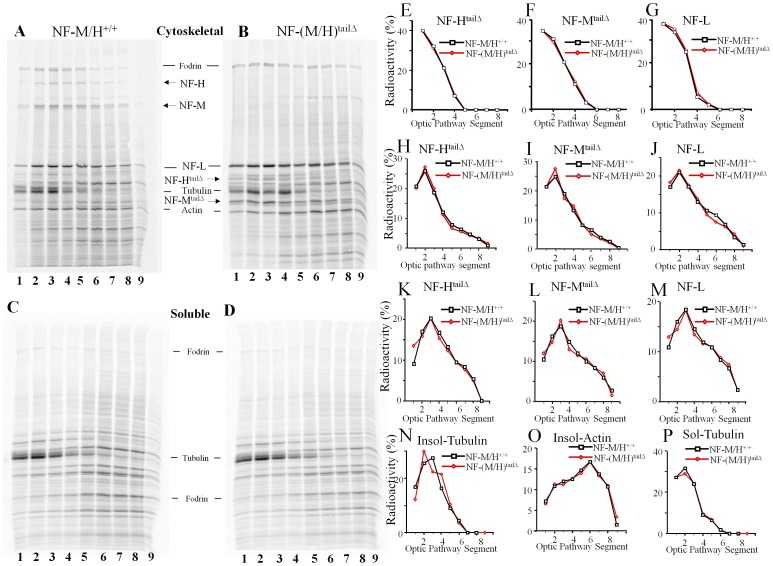
Absence of NF-H/M tails does not influence the rate of transport of NFs in optic axons. The speed and composition of slow axonal transport was determined by intra-vitreal injection of radiolabeled [^35^S]-methionine of 3–4-month-old WT (A, C) and NF-(H/M)^tailΔ^ (B, D) mice for 3, 7 and 14 days. Optic nerve proteins were fractionated into cytoskeleton (A, B) and soluble fractions (C, D) with a Triton X-100 containing buffer. Fractionated proteins were separated on 5–15% SDS-polyacrylamide gels, transferred to nitrocellulose membranes, and visualized by x-ray film and phosphorimaging. *(E–P)* Quantification of NF-subunits, tubulin and actin transport in optic nerves. (E, H&K)-NF-H&NF-H^tailΔ^; (F, I&L)-NF-M&NF-M^tailΔ^; (G, J&M)-NF-L; (N&P)-tubulin; and (O)-actin. (E-G)-3day; (H–J)-7 day; (A–D; K–P)-14 day transport.

NF-(H/M) subunit C-terminal tail domain hyperphosphorylation has been implicated in slowing of NF transport rate [31], [32], [39], [40], [41]. We analyzed slow transport of Triton-insoluble and soluble radiolabeled proteins in optic axons after pulse labeling retinal ganglion cell proteins of NF-(H/M)^tailΔ^ and WT mice by injecting [^35^S]-methionine intravitreously [37]. The ratio of total NF protein radioactivity relative to that of actin, serving as a control protein, was comparable in NF-(H/M)^tailΔ^ and WT optic axons at 3, 7 and 14 days. We observed no differences in the levels of radiolabeled NF proteins entering optic axons at these time points (Fig. 4A). Analyses of the distribution of labeled proteins along 8 consecutive 1 mm segments of the optic pathway showed that the fast moving edge of the NF transport wave advanced at the same rate in WT and NF-(H/M)^tailΔ^ optic axons by 3 days (Fig. 4E–G), 7 days (Fig. 4H–J) and 14 days (Fig. 4A–D, K–M) after isotope injection. The transport rate was constant during this time interval (0.14 mm/day), and similar to that previously reported [42]. Similarly, transport rates for tubulin (Fig. 4N&P), actin (Fig. 4O), and other cytoskeletal proteins (e.g., Fodrin, microtubule-associated protein 1A) (not shown) were also unaltered in NF-(H/M)^tailΔ^ mice at all of these time points.

### NF-H/M tail domains are a determinant of the long residence times of stationary neurofilaments in axons

**Figure 5:**
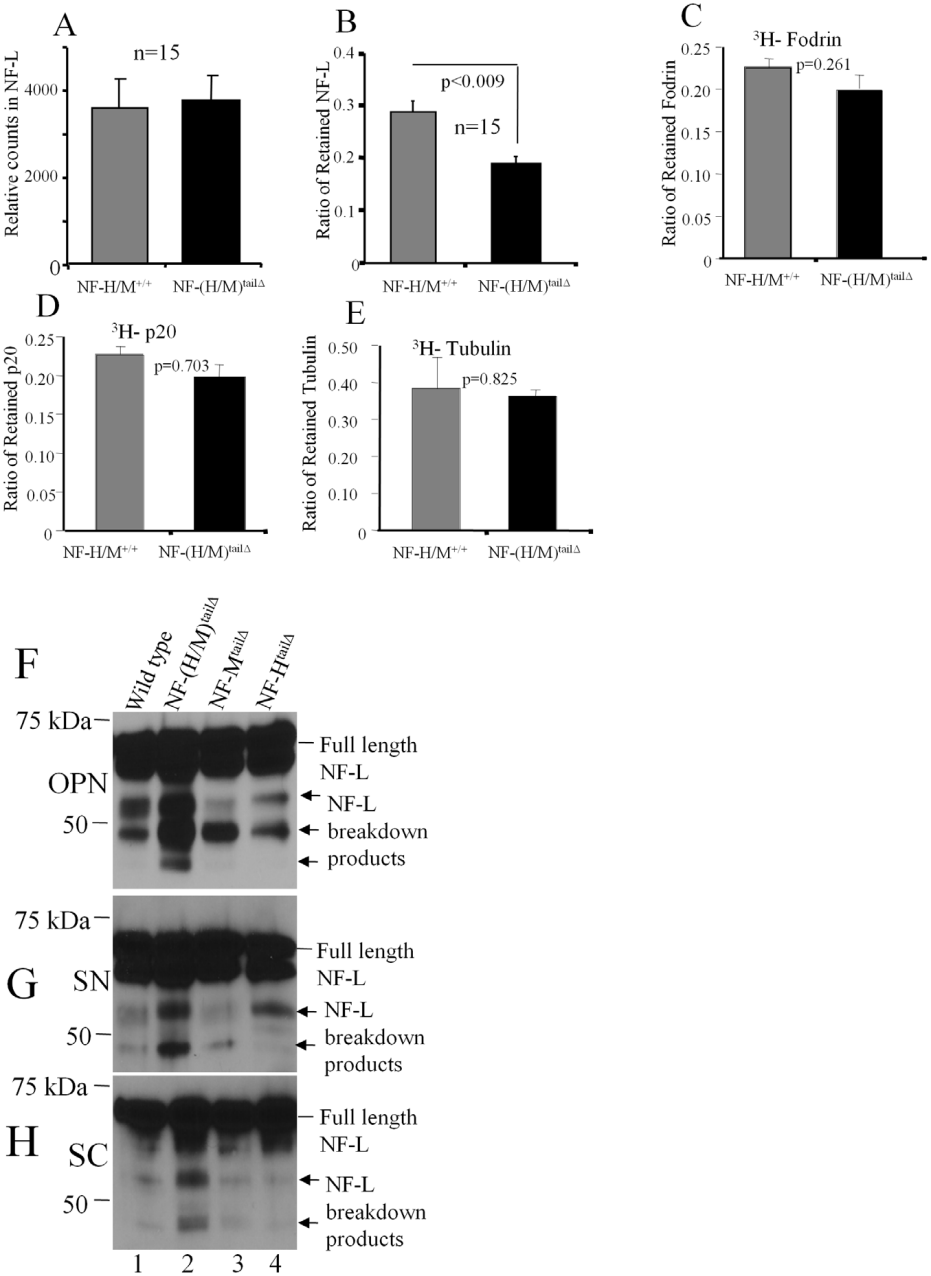
Increased turnover and degradation of NF-L in NF-(H/M)^tailΔ^ optic axons. (A) Unaltered NF-L synthesis in NF-(H/M)^tailΔ^ optic axons. (B). Decreased ratio of retained NF-L in NF-(H/M)^tailΔ^ optic axons indicate increased turnover of NF-L while the ratios of fodrin (C), p-20 (D), and tubulin (E) were not significantly altered. Increased NF-L degradation in optic nerve (F), sciatic nerve (G) and spinal cord (H) of NF-(H/M)^tailΔ^ mice as evidenced by generation of proteolytic fragments detected by immunoblots probed with NR-4 antibody (see the arrows in panels F–H). Error bars represent SEM in all experiments.

Using a second pulse-radiolabeling paradigm, we investigated the long term disappearance rate of NF proteins from a 8 mm length (“window”) of the optic pathway (optic nerve and tract) after injecting identical amounts of [^3^H]-proline intravitreously in large groups of WT and NF-(H/M)^tailΔ^ mice. Radioactivity associated with NF-L was measured at 14 days post injection – a time point when peak levels of pulse-radiolabeled NF proteins have entered the “window” and none has moved out [28]. These analyses showed that NF-(H/M)^tailΔ^ and WT mice exported similar levels of labeled NF-L into optic axons indicating that unaltered levels of newly synthesized NF-L and entry into axons in NF-(H/M)^tailΔ^ (Fig. 5A) despite having 3–6 fold lower number of neurofilaments along these axons (Fig. 2&3).

We have previously shown that radiolabeled NF proteins remaining in the optic axon “window” beyond 45 days after isotope injection are incorporated into a stationary NF network because the distribution of this population along axons does not change during the next 5 months [28], [37]. To assess the loss of this NF population in optic axons of NF-(H/M)^tailΔ^ and WT mice, we measured levels of radiolabeled NF-L at 90 days post injection in groups of these mice intravitreously injected with identical quantities of [^3^H]-proline. Our analyses showed that levels of radiolabeled NF-L were significantly decreased (32%, p<0.009) at 90 days but not 14 days, in NF-(H/M)^tailΔ^ optic nerves (Fig. 5E). This differential loss of NF-L in NF-(H/M)^tailΔ^ mice was selective because the ratios of fodrin (Fig. 5F), p20 (a 20-kDa protein, Fig. 5G), and tubulin (Fig. 5H) were similar in NF-(H/M)^tailΔ^ and WT optic axons.

The reduced axonal residence time for NF-L in NF-(H/M)^tailΔ^ mice was also associated with changes in the patterns of NF-L degradation (Fig. 5I–K). Immunoblots of total extracts (optic nerve, sciatic nerve, and spinal cord) from WT, NF-(H/M)^tailΔ^, NF-M^tailΔ^, and NF-H^tailΔ^ mice overexposed to highlight degradation products revealed increased levels of NF-L degradation fragments (55 to 45-kDa) in all 3 neuronal tissues from NF(H/M)^tailΔ^ mice compared to the patterns in the other mouse genotypes analyzed (Fig. 5I–K). These results indicate that the more rapid disappearance of NF proteins from optic nerves of NF-(H/M)^tailΔ^ mice is due to at least in part to increased NF proteolysis.

## Discussion

Our results demonstrate a critical function for the C-terminal domains of NF-M and NF-H subunits in stabilizing the axonal NF network by increasing its resistance to proteolysis, thereby expanding the content of neurofilaments in axons. The carboxyl terminal tail domains of NF-H and NF-M have previously been shown to form fine long cross-bridges that interconnect adjacent NFs and NFs and microtubules [21], [22], [43], [44], [45]. By means of a varied group of cytoskeleton-associated proteins [46] [23], [44] the NF network is interlinked to the microtubule, actin and spectrin cytoskeletal elements to establish a three-dimensional lattice in the axon. This stationary support structure, maintained by transported cytoskeletal elements or their precursors, serves as a platform for transport, reversible docking and organization of vesicular organelles and possibly other macromolecular complexes in axons [6] and provides the tensile support for axon caliber expansion required for electrical conductance along axons.

When the C-terminal domain of both NF-H and NF-M are eliminated in NF-H/M tail deletion mice, the NF cytoskeleton loses most of its cross-bridging and appears highly disorganized [47]. In normal axons, the neurofilament cytoskeleton is remarkably stable with NF subunits exhibiting exceptionally long half-lives of several months [27], [28], [37]. We observed that loss of the C-terminal regions had negligible effects on entry and transport of NFs in axons but increased the rate of disappearance of stationary NFs from axons leading to a markedly decreased NF content along optic axons. Although the factors that contribute to the turnover of the NFs are not clear, the unstable NF networks in NF-H/M tail deletion mice may be more prone to proteolysis in the axon by cellular proteases such as calpains. Calpains have been shown to be involved in turnover of NFs, particularly when they are relatively poorly phosphorylated at their C-terminal ends [48], [49]. Our study resolves the longstanding issue of whether C-terminal domains of NF-M and NF-H regulate the axonal transport of NF by demonstrating that NFs that lack these domains exhibit the same transport velocities along axons as NFs with intact physiologically phosphorylated C-terminal tails. Based on correlative evidence, it was widely believed that phosphorylation of NFs along their carboxyl terminal domains regulates the slow axonal transport of NFs [26], [31], [32], [39], [50]. *In vivo* slow axonal transport studies from NF-Hdeleted, NF-H^tailΔ^, and NF-M^tailΔ^ optic nerves indicate that neither loss of the entire NF-H subunit, nor the loss of the tail domains of NF-H or NF-M, alters slow NF transport [7], [21], [22], [34]. Due to the possible functional redundancy of these tail domains, it was thought that an intact C-terminal domain on either subunit could preserve putative transport functions when the C-terminal domain is lost on the other subunit. By eliminating both C-terminal domains, we showed here that regardless of any functional redundancy, transport is not affected by combined loss of these domains.

Although our evidence excludes a physiological role for C-terminal domains in regulating normal NF transport, it does not exclude the possible significant effect of pathological C-terminal tail phosphorylation in influencing NF transport. In vitro culture studies with GFP-labeled NF-H C-terminal KSP mutants that mimic hyperphosphorylation of NF-H C-terminal domain indicate that phosphorylation of NF-H KSP sites on the tail domain does slow the rate of axonal transport of NFs [41]. These overexpressed NF-H mutants that mimic hyperphosphorylation may represent pathological conditions that are observed in multiple NF transgenic lines and ALS mouse models. Hyperphosphorylated C-terminal regions of both NF-H and NF-M are observed in human ALS patients and in mouse models that exhibit ALS- like disease symptoms after NF overexpression [51], [52] or overexpression of mutant SOD1 [53]. In these transgenic mouse models, NF transport rates were significantly reduced suggesting that defective axonal transport of NFs may contribute to NF accumulation and other ALS-related pathological symptoms. NF-M and NF-L head domains are also relatively highly phosphorylated but most of these phospho-residues are short lived, disappearing as soon as NFs enter into the axon [54]. We speculate that some of the phosphorylation sites on these domains may regulate the NF axonal transport rate. Although phosphorylation on head domains is believed to prevent NF assembly in cell bodies, axonal transport studies using NF-L head domain phosphorylation mutants that mimic permanent phosphorylation of NF-L head domain in cortical neurons demonstrate that head domain of NF-L partly regulates NF axonal transport [55].

The results of this study, demonstrating dramatic non-uniform depletion of NF along NF-(H/M)^tailΔ^ axons despite unchanged transport rate, reinforce the notion that NF transport rate is not the principal determinant of NF content in mature axons, which is, instead the incorporation of transported NF into a large slowly turning over stationary NF network. We show here that the size of this stationary network is modulated at least in part by changes in the long half-lives of NF in this network, although the rate of incorporation of slowly transported NF into the stationary network cannot be excluded as an additional factor. The greater reduction of NFs at proximal levels is consistent with our original observations [37] that degradation of the stationary NF cytoskeleton takes place locally in the axon although the relatively small fraction of newly synthesized NF protein reaching the nerve terminals may be degraded there as described previously [56].
